# “Temperine” Contained Sufficient Alcohol to Make It Intoxicating, and Shippers Are Fined $100 and Costs*Office of Information, U. S. Department of Agriculture, Washington, D. C.

**Published:** 1913-10

**Authors:** 


### "Temperine" Contained Sufficient Alcohol to make it Intoxicating, and Shippers are Fined $100 and Costs.*

Notice of judgment, just issued by the Department of Agriculture, states that Herman Friedman, Paducah, Ky., has pleaded guilty to the misbranding of a "Temperance Drink" called "Temperine," and has been fined $100 and costs. Purporting to be a temperance drink, it was found on analysis to contain 2.77 per cent, by volume, of alcohol, sufficient, it was claimed, to make it intoxicating.

The label stated that it contained less than one-half of one per cent of alcohol. The product was shipped from Kentucky into

*Office of Information, U. S. Department of Agriculture, Washington, D. C.

Illinois. The defendant traded under the name of A. M. Laevison & Co.

